# Myelomeningocele, a congenital defect with severe form of spina bifida: a rare clinical image

**DOI:** 10.11604/pamj.2022.42.281.36395

**Published:** 2022-08-15

**Authors:** Ashna Gledina, Ranjana Sharma

**Affiliations:** 1 Department of Medical Surgical Nursing, Smt. Radhikabai Meghe Memorial College of Nursing, Datta Meghe Institute of Medical Sciences, Sawangi, Wardha, Maharashtra, India

**Keywords:** Myelomeningocele, spina bifida, split spine, congenital malformation, birth defect

## Image in medicine

Myelomeningocele is a severe form of spina bifida in which the spinal cord and nerves develop outside of the body and are contained in a fluid-filled sac that is visible outside of the back area. Spina bifida is a neural tube defect with average incidence of 1-2 cases per 1000 population with female to male ratio of 1.2: 1. These defects occur as a result to a teratogenic process that causes failed closure and abnormal differentiation of the embryonic neural tube. We report a case of 27-year-old multigravida, whose ultrasound findings showed lumbosacral myelomeningocele at 23^rd^ weeks. At 30-weeks gestation, preterm labor due to premature amniorrhexis and placental abruption, led to the emergency caesarean-section. A female child was born with birth weight of 520 gm, required immediate oxygen and incubator support. A clinical diagnosis of myelomeningocele, a severe form of spina bifida was made. Newborn was referred to neonatal intensive care for further management.

**Figure 1:**
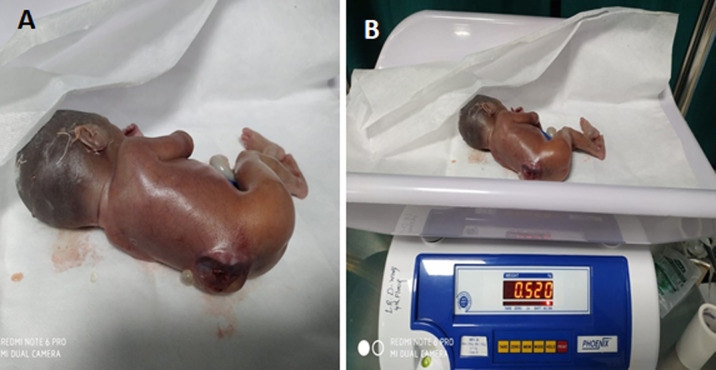
(A,B) myelomeningocele, open spina bifida with a sac of fluid comes through an opening in the baby’s back

